# Nurses' Experiences from Patient Safety Incidents of Hospitalized Children: A Qualitative Study

**DOI:** 10.1155/2024/1826514

**Published:** 2024-07-12

**Authors:** Haeyoung Lee, Yujeong Kim

**Affiliations:** ^1^ Red Cross College of Nursing Chung-Ang University, Seoul, Republic of Korea; ^2^ College of Nursing Research Institute of Nursing Innovation Kyungpook National University, Daegu, Republic of Korea

## Abstract

This qualitative study aimed at exploring nurses' experiences concerning patient safety incidents among hospitalized children in South Korea. From August 4 to 12, 2023, data were collected through in-depth individual interviews involving 14 clinical nurses. Employing thematic analysis, we identified 8 themes, which coalesced into three theme clusters: “challenges in pediatric patient safety nursing due to patient and caregiver characteristics,” “emotional changes in nurses following patient safety incidents,” and “sincere desire to prevent patient safety incidents in pediatric patients” The findings underscored that nurses experience significant burdens related to patient safety, emphasizing the necessity for robust support from caregivers, healthcare institutions, and national policies. Consequently, it is imperative to develop and implement programs and policies to foster a secure care environment for pediatric patients. Nurse managers and organizations must proactively design healthcare systems and related policies that prioritize safely protecting pediatric patients and nurses alike from patient safety incidents, considering the characteristics of pediatric patients and the experiences of the nurses caring for them.

## 1. Introduction

Ensuring patient safety in public healthcare remains a critical challenge globally, given its significant impact on mortality and disability rates [1]. In low- and middle-income countries, hospitals have witnessed approximately 134 million adverse events stemming from unsafe care [2]. Shockingly, these events contributed to 60% of all reported mortalities due to inadequate and substandard care [3]. Similarly, in high-income countries, on average, one in ten patients is reported to experience adverse events during hospitalization [4]. Patient safety encompasses a comprehensive approach, integrating cultures, processes, procedures, behaviors, and environments to mitigate risks and errors that could harm patients. It is currently a strategic priority for the healthcare field and is emphasized as a core aspect of national efforts towards universal health coverage [1].

Hospitalized children are especially vulnerable to severe, or even fatal, harm in the event of patient safety incidents. This vulnerability stems from their immature physical and physiological functions, as well as their limited ability to assess and manage risky situations [5]. Moreover, children are at a stage of rapid growth and development, and patient safety incidents at this stage can have severe, long-term effects on not only physical development but also psychological, cognitive, and social development [6]. Since nurses are the closest professional group providing direct care to hospitalized children, they are in an important position to improve patient safety [7]. While caring for patients, nurses experience many different patient safety incidents, including medication errors, falls, and bedsores [8]. Despite diligently performing their duties, these incidents can be unpredictable and persistently occur. It is imperative to delve deeper into nurses' firsthand experiences with patient safety incidents in clinical settings through comprehensive research to foster safer hospital environments and systems tailored to pediatric patients.

In our examination of both domestic and international literature concerning patient safety incidents involving hospitalized children, we came across studies that focused on family perceptions or experiences related to these incidents [9–11]. Additionally, research has delved into the perspectives of physicians, nurses, and patient care technicians concerning collaborations with parents to ensure patient safety [12]. Moreover, investigations have explored the relationship between medication errors and job satisfaction among nurses in pediatric wards [13]. However, there is a notable gap in research specifically addressing the varied experiences of pediatric nurses who directly care for pediatric patients, as well as pinpointing areas of concern and improvement in pediatric patient safety.

In the case of South Korea, the total fertility rate in 2022 is 0.778, which is half of the OECD average of 1.59, the lowest among OECD countries [14]. In addition to the rapid decline in the number of newborns, the cost of pediatric treatment is very low. The lack of doctors and long waiting times cause a lot of rudeness, verbal abuse, and legal disputes from patients' parents against medical staff, making it the worst treatment environment for doctors and nurses [15]. As a result, doctors avoid treating children, and the pediatric care system in South Korea is collapsing [16]. Therefore, it is necessary to analyze the experiences of pediatric patient safety accidents that occurred in such a heavy workload and stressful working environment and establish policies to create an optimal working environment for pediatric nurses to improve pediatric patient safety.

In the present study, we applied qualitative research methods to delve deeply into the experiences of nurses regarding patient safety incidents involving pediatric patients. Our objective was to identify challenges, obstacles, and potential strategies to enhance the management of patient safety incidents as perceived by the nurses. We anticipate that our findings will help contribute to the formation of hospital environments adept at preventing and addressing patient safety incidents in pediatric care. Furthermore, we aspire to influence the development of healthcare policies that prioritize the safety and well-being of both patients and nurses.

## 2. Materials and Methods

### 2.1. Study Design

This was a qualitative study collecting and analyzing data from in-depth interviews, intending to comprehensively understand and explore nurses' experiences of patient safety incidents in hospitalized children.

### 2.2. Participant Selection and Investigator Preparation

The inclusion criteria for participants in this study were specific: We sought nurses currently employed at healthcare institutions with at least 6 months of nursing experience. Additionally, these nurses must have encountered patient safety incidents involving pediatric patients they cared for within the past three years. Importantly, the incidents should have involved patients aged 15 years or younger at the time of hospitalization. The sole exclusion criterion was a refusal to have the interview recorded.

The researchers posted information about the study objectives and methods to an online community for nurses in Korea. People who wanted to participate could use a link or a QR barcode to access an online site for study participation. Visitors to the website could review the comprehensive study details and provide their contact details if they chose to participate voluntarily. Subsequently, the investigators reached out to these individuals to elucidate the study's purpose and arrange interview sessions. Interviews were conducted until reaching theoretical saturation, meaning no new significant insights emerged from participants' narratives. Ultimately, the study involved 14 participants.

Given that the main instruments for qualitative research are the researchers themselves, to ensure reliability and train their ability to analyze results, the researchers gave talks and lectures on qualitative research in graduate courses and at related academic conferences. The researchers also have direct experiences of conducting qualitative research several times. During our tenure at the hospital, the researchers worked at a quality and patient safety center that performs activities to analyze and improve patient safety incidents. During this time, they analyzed the causes of patient safety incidents, interviewed healthcare workers, patients, and caregivers to make improvements, and accumulated practical experience understanding the interview content. The corresponding author also has extensive experience in pediatric nursing for 5 years working as a nurse in a pediatric ICU. We made efforts to minimize the effects of researcher bias or opinions on the interviews and results analysis and to maximally reflect the experiences and opinions of the participants.

### 2.3. Ethical Consideration

Before conducting the study, we received approval from the institutional review board at Kyungpook National University (no. 2023-0365), where the participants were affiliated. Subsequently, we initiated the data collection process. Before conducting the interviews, the researchers thoroughly explained the study objectives, procedures, and methods to the participants. Importantly, participants were assured of their right to withdraw from the study at any time. The researchers explained that data collected from the participants would not be used for anything other than research purposes. Additionally, we clarified that all interviews would be recorded with strict confidentiality measures in place to safeguard participants' privacy. Furthermore, we assured participants that their involvement would incur no adverse consequences. Individuals willing to participate then submitted their online consent forms online before proceeding with the interview. The interview data were processed directly by the researchers to protect the participants' privacy; the data were coded and then stored in the laboratory. The participants were sent a mobile coupon as a token of appreciation.

### 2.4. Data Collection

The individual in-depth interviews with participants were conducted between August 4 and August 12, 2023. Given the prevailing COVID-19 circumstances, we opted for remote interviews facilitated through Zoom. To prevent missing or erroneous data, only audio recordings were made with explicit consent, ensuring no video recordings were captured to prevent the disclosure of personally identifiable details. Each participant was interviewed once, with each interview taking between 60 and 100 minutes. The questions in the interview guide were composed by two researchers who had been responsible for analyzing patient safety incidents at a healthcare institution and who had ample experience in interviewing healthcare workers. The questions were semistructured, and the interview guide was provided in Supplementary 1. The main question for the in-depth interviews was, “What experiences have you had relating to patient safety incidents affecting hospitalized children you were caring for?” Additional questions included the following: “If the cause of the incident was due to healthcare workers, caregivers, or the hospital, what was the cause?” “What do you find most difficult about patient safety nursing for pediatric patients?” and “What is needed to prevent and ameliorate patient safety incidents for pediatric patients?” Questions were asked sequentially, depending on the flow of the conversation, making efforts to extend the conversation without interrupting the participant. The participants' speech was transcribed word-for-word, and nonverbal expressions, such as silence and sighs, were also recorded during the interview.

### 2.5. Data Analysis

The recorded data in the analysis, including the transcriptions of the interview recordings and the researchers' notes taken during the interview, amounted to 176 pages of A4 paper. Postinterview, the recorded content was promptly transcribed within a day. Two researchers performed data analysis using a thematic analysis approach after all the interviews had been completed. The researchers reviewed and supplemented the analysis through discussions to produce the final analysis. The thematic analysis used in this study was performed in 6 stages, following the method of Braun and Clarke [17]. (1) The transcripts of the recordings were read repeatedly to achieve familiarity with the data; (2) the meaningful content in the transcripts was coded, and content corresponding to each code was combined; (3) potential themes were generated by grouping codes, and all data related to each theme were organized; (4) the relationships between the themes and coded data were reviewed, and similar themes were grouped repeatedly to define theme clusters; (5) each theme was given a distinctive name; and (6) examples were identified that could explain each theme, and the report was written.

### 2.6. Validity and Rigor

We aimed to ensure the rigor of this qualitative study based on the factors proposed by Sandelowski [18]: credibility, auditability, fittingness, and confirmability.

For credibility, at the participant selection stage, we recruited participants who had previous experiences of patient safety incidents affecting hospitalized children, and those who could describe their experiences. Through open questions and a comfortable atmosphere, we created an interview environment that would allow participants to express their opinions freely. Moreover, by transcribing interviews promptly within a day, we aimed to minimize omission and distortion of the interview content. During data analysis, we aimed to derive credible results through thorough discussions between the researchers.

Addressing auditability, we aimed to describe the study objectives and methods in detail and aid readers' understanding.

To satisfy the criteria for fittingness, we described the participants' general characteristics. In addition, we collected data until the saturation point, when participant's statements were repeating, and we could no longer derive any new content.

Finally, to ensure confirmability, we made efforts to minimize the researchers' bias, maintaining a neutral stance throughout the research process. This approach safeguarded against any undue influence of researchers' feelings or experiences on participants' responses.

## 3. Results

The general characteristics of the participants are presented in Table 1. The participants were all female, with a mean age of 31.6 ± 5.5 years. Specifically, seven participants (50.0%) fell within the 20- to 29-age bracket, while five participants (35.7%) were aged between 30 and 39 years. Regarding the type of healthcare institutions, 12 participants (85.7%) were affiliated with tertiary hospitals. In terms of departmental roles, 10 participants (71.4%) served in general wards, three (21.4%) worked in intensive care units, and one (7.2%) was stationed in the emergency room. The mean nursing experience was 8.0 ± 5.7 years, and three participants (21.4%) were parents themselves.

During the analysis of the interview content, 199 codes were generated in stages 1 and 2, and mutually related codes were classified to form 8 themes in stage 3. In stages 4 and 5, the themes were structured into three theme clusters with more comprehensive meanings: “challenges in pediatric patient safety nursing due to patient and caregiver characteristics,” “emotional changes in nurses following patient safety incidents,” and “sincere desire to prevent patient safety incidents in pediatric patients” (Table 2).

### 3.1. Challenges in Pediatric Patient Safety Nursing due to Patient and Caregiver Characteristics

#### 3.1.1. Risk due to Difficulties Communicating with Pediatric Patients

The participants had experienced difficulties precisely identifying symptoms in pediatric patients because children have difficulty expressing themselves clearly or are afraid of healthcare workers, while infants are incapable of communication. When a pediatric patient cries or complains of discomfort, it is difficult for nurses to tell whether it is due to pain, or if the child is simply whining. This can delay diagnosis, examinations, or treatment, amplifying the perceived urgency and potential risk of the situation. Given the inherent nature of childhood marked by curiosity, pediatric patients frequently engage in playful and sometimes reckless behaviors. This can include attempting to interact with medical equipment, removing dressings from injection sites, or even jumping on beds or climbing on IV poles despite being cautioned about the potential risks of falls. Such behaviors escalate the likelihood of patient safety incidents.

“Most children can not easily express themselves verbally, so you do not know if they are crying because it hurts. Often, even if I want to be proactive and help the patient quickly, I have to take a moment to think again. Those moments are difficult. It would be much better if they could tell us where they were hurting, but as the nurse, you have to test one thing at a time, and that takes a long time” (Participant 3).

“Children do not stay still just because you tell them to. They do not take the hospital seriously, and they run around all over the place playing however they like, pressing infusion pump buttons and turning IV regulators” (Participant 6).

#### 3.1.2. Risk due to Excessive Workload

The participants highlighted that it was difficult to find the vein when placing an IV catheter in pediatric patients. Furthermore, the procedures took a long time due to a lack of cooperation, especially if patients were crying or moving, and they often required help from other people. Newly graduated nurses, lacking experience, frequently encounter difficulties in delivering treatments to pediatric patients, leading to errors that require intervention from more seasoned nurses. Additionally, tasks like changing hospital gowns or bedding for pediatric patients demand more time compared to adult care, intensifying the workload. Moreover, because children require smaller doses of medication, the dose needs to be calculated precisely, and when heavy workloads make it difficult to practice safe nursing, there is a high risk of errors, which can lead to dangerous situations for the patient. This results in a greater sense of burden and danger for nurses.

“When children try to grab the IV, one person each needs to hold the arms and legs, so everyone has to stop what they are doing and help hold the IV. It is difficult to find the vein in children, and they cry and move around, and so you have to wrestle with them for twenty minutes. For all that time, nobody working on the ward can get anything else done” (Participant 6).

“When giving medication to adults, the prescription is 1 vial, so you can just give them the whole vial. For children, you have to calculate the dose accurately, and of course, you have to hand up the infusion pump, which takes more time and is something you have to worry about. In children, the consequences of me making even a small mistake can be very severe, and so it is a major burden for nurses. We should check one more time and be meticulous, but it is easy to miss something when we're so busy” (Participant 13).

#### 3.1.3. Stress due to Caregivers' Sensitivity

Participants indicated that caregivers, despite recognizing the demanding nature of nursing duties and the critical needs of other patients, often prioritized their own child's care. Consequently, some caregivers expressed anger, frustration, and impatience when they felt their child was not attended to promptly. In the ICU, caregivers would constantly call with inquiries even beyond designated visiting hours. Such interactions, coupled with caregivers occasionally eavesdropping on conversations, contributed to heightened stress levels among nurses. Due to these challenges, many nurses avoid working in pediatric wards, and new pediatric nurses also complain of high levels of stress.

“The sensitivity of caregivers is probably a difficulty that only pediatric nurses have to experience. Lately, people aren't having many children, and when it is the parent's only child, it is really precious. Even though I've told the caregiver that I have an urgent situation or I have to give CPR to another patient, they shout at me, asking me to change the sheets. They can see for themselves that it is an emergency, but a lot of them get too sensitive about trivial things. The caregivers are far more sensitive than those in an adult ward” (Participant 7).

“A lot of nurses refuse to work in the pediatric department. That's because there are so many complaints from caregivers. Everyone avoids working in the pediatric wards because the caregivers complain about everything, even down to the nurses” tone of speech. Experienced nurses may be able to somewhat control rude or sensitive caregivers, but new nurses are reduced to tears and say that controlling the caregivers is the hardest part (Participant 3).

### 3.2. Emotional Changes in Nurses following Patient Safety Incidents

#### 3.2.1. Getting Swept Up in Negative Emotions

Following patient safety incidents, such as falls or medication errors, participants reported feeling surprised by the incident and also sorry that they could not care properly for the patient as their nurse. They were also worried or fearful of the patient's condition worsening due to the safety incident and felt guilty about their error. Moreover, strained relationships with caregivers added to their sense of burden, making communication challenging. On the other hand, even though it was a patient safety incident caused by the mistake of a child or parents, nurses wrote the incident report, and some participants felt wronged by the fact that the nurse was held responsible for the cause of the accident blaming it on the nurse's negligence and lack of explanation.

“I felt a sense of shame, thinking thoughts like, “I made a mistake to the child,” “What if something happens to the baby because of me?,” “Why did I make another mistake?” I felt like I had made an enormous mistake, and I felt like I was tiny” (Participant 12).

Writing a fall report is very stressful and difficult for nurses. It feels like it is placing too much of the responsibility on the nurse. I feel harshly treated because it makes it so that the fall is the fault of the primary nurse” (Participant 1).

#### 3.2.2. Trying Even Harder to Prevent Accidents

Following a patient safety incident, the participants worked hard to follow patient safety principles, such as removing the IV site fixation dressing and checking for redness and swelling even if there is no complaint of pain, and repeated education on the risk of falls and the importance of prevention, even though they were busy. The participants performed their rounds more intensively to prevent new patient safety incidents and recurrence. They also continually provided education, explaining safety rules and their importance to improve caregivers' perceptions of patient safety.

“Since it is all we can do to explain about safety, we try to keep reminding the caregivers. And we really did a lot of rounds. Not only the on-duty nurse, but also the nurse managers, and the nurse practitioners visited the patient multiple times” (Participant 8).

“Since the patient safety incident, I made efforts to follow the basic rules more precisely. I thought I should have checked the IV site, even if the child was sleeping. Since then, I explained a little more to the caregiver that, because it is an important and somewhat dangerous drug, they need to check the injection site, even if the child wakes up, and I also check more often” (Participant 13).

### 3.3. Sincere Desire to Prevent Patient Safety Incidents in Pediatric Patients

#### 3.3.1. Improving Safety Awareness of Caregivers

Participants observed that caregivers frequently overlooked patient safety concerns. They expressed concerns about caregivers leaving infants unattended on beds while attending to other matters outside the ward. Additionally, caregivers often seemed distracted and engrossed in their phones or television screens, potentially compromising the child's safety within the ward. Participants emphasized the need for caregivers to prevent children from engaging in hazardous behaviors and underscored the importance of imparting discipline. Recognizing that caregivers are ideally positioned to identify shifts in a patient's condition, participants highlighted their pivotal role in effectively communicating the child's status to healthcare professionals.

“Hospitals are unfamiliar environments for children, with a high risk of falls. Sometimes caregivers will lie down and play with their phones or watch TV or go in and out of the ward to take a phone call. It's actually really dangerous to just leave a child alone. I think they feel like nothing could happen in a hospital” (Participant 6).

“Actually, the caregivers know better than me how the child is different from usual. It would be good if caregivers would tell the healthcare workers immediately if there was anything that seemed strange or there seemed to be a problem, and if they would ask us what had happened. Since there are many things we miss, these questions would provide us with an opportunity to check the patient again. It's surprisingly uncommon for caregivers to ask questions like, “Why is this the case?” when visiting. Usually, they just say something like, “Please take good care of him/her” (Participant 14).

#### 3.3.2. Improving Pediatric Care-Related Infrastructure and Policy Support

Participants highlighted that the diminishing number of pediatric residents in Korea over the last several years has resulted in an increased workload for nurses, as they assume tasks typically handled by pediatricians in tertiary hospitals. This shift has intensified work pressures. Furthermore, the participants felt that patients' health was threatened by the gap in pediatric treatment due to the closure of pediatric hospitals and clinics. In addition, participants emphasized that caring for children entails more nursing responsibilities compared to adult patients. Patient safety incidents were attributed to the existing shortage of nursing staff. Specifically, new nurses have to care for the same number of patients despite lacking the required knowledge and skills, which further increases the risk of patient safety incidents. Participants expressed concern about the demanding workload of all nurses, such that newer ones found it challenging to seek or obtain assistance when needed. The participants reported the need for national policies to improve support for pediatric healthcare workers, including doctors and nurses, and to increase the fees for pediatric healthcare. In addition, given the characteristics of pediatric patients, they reported the need for support to further develop medical devices and materials to prevent patient safety incidents, urging their integration into healthcare institutions.

“We haven't had any new pediatric residents for 2 years, so the 3rd and 4th-year residents are continuing to be on duty on alternating days. As a result, the excessive workloads of pediatric doctors are getting worse. This means that doctors' work is being passed on to nurses. The lack of doctors is ultimately leading to excessive work for nurses. Blood collection was usually done by doctors, but now it is being done by nurses. Our time spent directly caring for patients has decreased, and I'm worried that this might lead to more patient safety incidents in pediatric wards in the future” (Participant 7).

“For medical materials as well, you can get by with just one or two sizes for adults, but you need to prepare all different sizes for children. It would be nice if we had all children's beds as well, because of falls, but nothing changes when we ask, because they are too expensive. Because children might try to press buttons on medical devices for fun, we need to bring in more secure devices, such as devices with locks” (Participant 3).

#### 3.3.3. Improving Support for Patient Safety Activities and Culture within the Hospital

Participants proposed that creating educational videos within hospitals to instruct pediatric patients and their caregivers about falls and general patient safety could prove highly effective in both education and incident prevention. Additionally, they believed that distributing patient safety-related pamphlets or campaigns could enhance caregivers' awareness of patient safety. In addition, participants wanted parents to understand the poor and busy working environments of nurses, urging for mutual respect. They also hoped that a positive patient safety culture would be established that would move away from the negative culture that focuses on criticizing and punishing healthcare workers who cause patient safety accidents. Instead, it would focus on improving the work environment to prevent future patient safety incidents. The participants also reported the need to strengthen activities and support for patient safety at the hospital level, such as asking the hospital to make a pediatric IV team to accurately and safely secure IVs in pediatric patients and to prevent vasculitis, which is a common complication among children.

“If we made short educational videos about patient safety and had the patient and caregiver watch these before admission, I think it would help with understanding when we explain. I think it would help if there were fall videos suited for children. Children can concentrate and watch videos on YouTube or other platforms. It would be easier for nurses to explain and also improve patients' awareness” (Participant 5).

“They blame everything on the nurses. Even if the doctor gave the wrong orders, they say it is the nurse's fault because they should have caught it. Nurses are busy and having a hard time because we have to see so many patients, but they do not think of that. When you are this busy, mistakes are inevitable, but they do not think about our circumstances and they only talk about the mistake. I wish [caregivers] would understand our situation and trust and listen to the healthcare workers” (Participant 14).

## 4. Discussion

In this study, we comprehensively explored nurses' experiences of patient safety incidents affecting hospitalized children. After analyzing the content of interviews with the nurses, we derived and discussed 3 theme clusters: “challenges in pediatric patient safety nursing due to patient and caregiver characteristics,” “emotional changes in nurses following patient safety incidents,” and “sincere desire to prevent patient safety incidents in pediatric patients.”

In the first theme cluster, participants expressed the sense of burden felt by pediatric nurses who always have to be extremely careful to try to prevent patient safety incidents while caring for pediatric patients. In addition, when caring for infant patients, clear communication is impossible, and cooperation during treatment is difficult, often resulting in prolonged treatment durations and heightened nurse workload. Furthermore, there is a considerable burden associated with the need to precisely calculate medication doses relative to body weight for children; however, sensitive reactions from caregivers who often prioritize their child's needs exclusively further exacerbate the stress of nurses. This aligns with findings from a prior study on pediatric nurses [19], where family caregivers sometimes exhibited behaviors, such as prioritizing their own child's needs to the exclusion of respecting nurses or even resorting to derogatory language. Such interactions elicited negative emotions from nurses and introduced ethical dilemmas when these sentiments impacted patient care. Given that children are inherently more vulnerable and susceptible to anxiety and fear than adults, it underscores the imperative for pediatric nurses to exhibit heightened patience, expertise, and an unwavering commitment to patient safety [20]. In China, the combination of a demanding workload and challenging interactions with discerning parents has been pinpointed as contributing factors to heightened work stress, burnout, and turnover rates among pediatric nurses, with a notable 40% of pediatric nurses expressing job dissatisfaction [21]. Family-centered care is a core aspect of pediatric nursing care, with nurses respecting the dignity of the child and their family and providing care based on partnerships [22]. However, for patients to receive the highest level of safe nursing, it is essential for the patient's parents also to try to understand and respect the difficulties of nurses and make efforts to form positive partnerships with the nurses. Moreover, there is a need to provide opportunities and compensation to improve pediatric nurses' workload, reduce work stress, and encourage continual career development [21].

In the second theme cluster, nurses reported being consumed by negative emotions, including not only surprise but also sorrow towards the patient, concern and fear about the patient's condition, and guilt about their own mistakes. However, they also worked even harder to follow patient safety principles after an incident, went on rounds more often to prevent recurrence, and continued education to explain the importance of patient safety to caregivers.

Since a hazardous accident can lead to severe injury or even death, the primary victims of such accidents are the patient and their family. Nevertheless, healthcare workers affected can also experience psychological shock and emotional distress and thus are recognized as second victims of these accidents [23]. The second victims of patient safety incidents show emotional responses, including guilt, anger, despair, psychological stress, and fear, and have even been reported to show physical symptoms, such as fatigue and insomnia, as well as divergent behaviors [24]. Some nurses have even experienced difficulties sleeping or eating [25]. These responses by second victims can lead to harmful outcomes in the personal and professional lives of healthcare workers, including posttraumatic stress disorder, turnover, and suicide [26]. Nurses experience more negative emotions compared to physicians [27], and this can lead to a worsening cycle by inducing burnout and depression, making nurses further unable to provide appropriate care [28]. Specifically, when nurses continue working without properly addressing the emotional stress stemming from a patient safety incident, it can escalate the risk of subsequent accidents. Moreover, improper healthcare provision can have additional effects on patients, other healthcare workers, and the overall reputation of the healthcare institution [29]. The negative emotions experienced by nurses after a patient safety incident can gradually improve. However, the lingering impact of such incidents can remain deeply distressing for an extended period [7], underscoring the urgency for timely resolution. As such, healthcare institutions need to ascertain the current state of second victims, explore related factors, and prepare individualized interventions for second victims depending on their symptoms [7]. Understanding the unique requirements of healthcare professionals is crucial when crafting targeted support programs for nurses affected by patient safety incidents [30]. Notably, distress experienced by these second victims can significantly influence work-related outcomes, including intentions to leave their positions and increased absenteeism; organizational support has also been reported to show a full mediation effect [26]. Therefore, to reduce turnover and absenteeism among nurses who have experienced patient safety incidents, it will be necessary to proactively develop support measures at the organizational level.

The third theme cluster centered on the “sincere desire to prevent patient safety incidents in pediatric patients.” Nurses often perceived the risk of patient safety incidence while observing caregivers who were not paying sufficient attention to the patient. Following patient safety incidents, there is a prevalent tendency among caregivers to place blame on the nurses, with organizations also primarily holding the staff accountable. Nurses articulated a strong desire for acknowledgment and respect from caregivers, coupled with the establishment of a robust patient safety culture within their institutions. Many nurses conveyed a sense of disappointment with the existing conditions. The majority emphasized the urgency of bolstering nurse staffing levels to enhance patient safety. They also underscored the necessity for national policies aimed at augmenting pediatric healthcare fees and fortifying support systems for healthcare professionals, encompassing both physicians and nurses. Additionally, there is a pressing call for organizational and political backing to facilitate the creation and effective deployment of medical devices and materials tailored to the unique needs of children. Within healthcare institutions, nurses advocated for a range of initiatives, including comprehensive patient safety education to heighten caregivers' awareness and the establishment of specialized pediatric IV teams.

As part of its program for patient safety, the World Health Organization (WHO), recognizing the roles and values of patient participation, has demanded cooperation and partnership between patients, family, healthcare providers, and policymakers in constructing safer healthcare systems and has encouraged patients to be at the center of healthcare services [31]. The Joint Commission has also suggested patient and family participation as one of 7 types of evidence for safe, high-quality care [32], and patient participation is known to be a means of preventing healthcare errors [33]. Patient participation involves fostering an environment that encourages the active participation of patients, families, and caregivers in their care to improve the safety, quality, and patient-centricity of healthcare services [34]. Healthcare institutions have the potential to promote this active patient involvement as a strategic measure to safeguard patients' health and well-being [35]. According to previous studies, patient participation in nursing is essential to guarantee high-quality care [36]. This active involvement provides important information for diagnosis and treatment and can potentially help to improve healthcare systems [37]. Because treatment-related decisions for pediatric patients are made by their caregivers, in this respect, promoting caregiver participation will help to prevent patient safety incidents, improve caregivers' safety awareness, and help to achieve safe nursing.

In a study investigating healthcare workers' perspectives on pediatric patient safety culture [5], findings revealed that aspects like organizational continuous-learning improvement and feedback and communication on errors were notably strong among healthcare professionals, including nurses. However, areas such as management support for patient safety and adopting a nonpunitive approach to errors were perceived weakly. This suggests that while there are robust communication and feedback mechanisms in place following patient safety incidents, there is a distinct need for enhanced support and fostering a nonpunitive culture for healthcare workers. This is consistent with our findings, with the participants in our study reporting that, in the event of a patient safety incident, both caregivers and the organizations only blamed individual nurses for the accident, and the participants were disappointed at the lack of respect from caregivers and the failure to establish a patient safety culture within organizations.

Most of the nurses who participated in the study complained of the need to increase nurse staffing for patient safety and emphasized the need for national policies to raise pediatric healthcare fees and improve support for pediatric healthcare workers. In 2021, among public healthcare workers in South Korea, the number of clinical physicians was 2.6 physicians per 1,000 population, which was very low compared to the OECD average of 3.7 physicians per 1,000 population, and the number of clinical nurses, at 8.8 nurses per 1,000 population, was also lower than the OECD average of 9.8 nurses per 1,000 population [38]. The so-called pediatric department “open-run,” where patients are waiting outside the doors at opening times for pediatric clinics, has become an everyday reality for parents [39]. Amidst the current climate of low birth rates in South Korea, healthcare fees for pediatrics are very low, and there is a high risk of legal disputes due to healthcare accidents, causing future residents to avoid applying to pediatric departments, thus threatening a collapse of the pediatric emergency care system [16]. Healthcare staff shortages are exacerbating workloads, which increases the risk of safety incidents; therefore, there is an urgent need for basic corrective measures.

Alongside national policies, nurses also reported the need for organizational support, such as the development and provision of medical devices and materials suited for pediatric patients. Nurses demanded specific activities and support for patient safety, such as improving patient safety education as a means of enhancing safety awareness among caregivers and forming specialized pediatric IV teams. It is critical to develop and implement safety-related education to prevent patient safety incidents from occurring at hospitals [7]. Since fall prevention education was effective at improving fall-related knowledge and preventive behaviors in caregivers of hospitalized children [40], we expect that the development and application of caregiver-targeted educational programs will help improve caregivers' awareness of patient safety. The preparation and implementation of these specific interventions could promote caregiver cooperation and accelerate the establishment of a patient safety culture in healthcare institutions.

## 5. Conclusions

In this study, we explored the experiences of nurses regarding patient safety incidents affecting hospitalized children, uncovering their sincere desire to prevent such incidents. It is imperative to devise organizational and policy initiatives that prioritize safeguarding both hospitalized children and nurses from patient safety incidents, incorporating the insights gleaned from this study. Within healthcare organizations, fostering a culture rooted in mutual understanding and respect is paramount. Fundamental steps include increasing nurse staffing, enhancing pediatric medical infrastructure, and formulating supportive policies. In this regard, nursing administrators and healthcare institutions must collaborate to strategize and advocate for pertinent policies at the organizational level.

## 6. Implications for Nursing Management

Considering the characteristics of patient safety incidents in children, caregivers' trust and respect for nurses, as well as their cooperation and participation in the patient care process, are crucial. Therefore, specific strategies are required to strengthen patient safety activities within hospitals. These strategies include educational programs aimed at improving caregivers' awareness and understanding of patient safety. Additionally, developing and implementing psychological support and counseling services, as well as peer support programs, can assist nurses in mitigating and overcoming the negative emotions associated with patient safety incidents.

Providing continuous education programs to enhance nurses' work efficiency and job satisfaction and establishing clear career paths for the professional development of pediatric nurses are critical considerations for organizational managers. Given the current characteristics of Korean society, such as having the world's lowest birth rate and the increased workload burden consequent on the decrease in pediatric specialists and infrastructure, it is imperative to devise long-term strategies to reduce the workload of pediatric nurses and safeguard them from patient safety incidents. This includes policy support for expanding the number of nurses and enhancing the infrastructure of pediatric hospitals.

A comprehensive set of policies should be developed to address the specific challenges facing pediatric nurses in the context of Korea's extremely low birth rate. These policies should support pediatric nurses by mandating rest periods, ensuring safe staffing ratios, and providing professional development opportunities. By integrating these innovative actions with context-specific insights, it will be possible to formulate comprehensive measures that effectively support pediatric nurses. Although Korea's low birth rate presents unique challenges, the strategies developed could offer valuable lessons for other countries facing similar issues in pediatric care.

To this end, active intervention and cooperation from nursing managers and policymakers are essential. Their involvement is crucial in the development and implementation of these policies and strategies, ensuring that pediatric nurses receive the needed support to deliver safe and effective care to their patients.

## Figures and Tables

**Table 1:** General characteristics of the participants.

ID	Sex	Age (yr)	Hospital	Department	Nursing career (yr)	Having children
1	F	28	General hospital	Ward	2.4	No
2	F	24	Tertiary hospital	Emergency room	1.5	No
3	F	40	Tertiary hospital	Ward	18.0	No
4	F	28	Tertiary hospital	Ward	2.5	No
5	F	34	Tertiary hospital	Ward	10.2	No
6	F	38	General hospital	Ward	16.5	No
7	F	30	Tertiary hospital	Ward	5.8	No
8	F	26	Tertiary hospital	Ward	4.3	No
9	F	35	Tertiary hospital	Intensive care unit	11.3	Yes
10	F	41	Tertiary hospital	Ward	15.0	Yes
11	F	29	Tertiary hospital	Ward	8.0	No
12	F	26	Tertiary hospital	Intensive care unit	0.8	No
13	F	34	Tertiary hospital	Ward	10.3	Yes
14	F	29	Tertiary hospital	Intensive care unit	5.0	No

**Table 2:** Nurses' experience related to patient safety incidents of hospitalized children.

Theme clusters	Themes
Challenges in pediatric patient safety nursing due to patient and caregiver characteristics	Risk due to difficulties communicating with pediatric patients
	Risk due to excessive workload
	Stress due to caregivers' sensitivity

Emotional changes in nurses following patient safety incidents	Getting swept up in negative emotions
	Trying even harder to prevent accidents

Sincere desire to prevent patient safety incidents in pediatric patients	Improving safety awareness of caregivers
	Improving pediatric care-related infrastructure and policy support
	Improving support for patient safety activities and culture within the hospital

## Data Availability

The data used to support the findings of this study are available from the corresponding author upon reasonable request.

## Supplementary Materials

Supplementary Materials Supplementary 1 include the interview guideline of this manuscript.
