# Supplementary material for: Nurses' Experiences from Patient Safety Incidents of Hospitalized Children: A Qualitative Study
Source: J Nurs Manag. 2024 Jul 12;2024:1826514. doi: 10.1155/2024/1826514 (PMC11919116; doi:10.1155/2024/1826514)
Supplement: Supplementary Materials — Supplementary 1 include the interview guideline of this manuscript. [file 1826514.f1.doc]

**인터뷰 가이드라인(Interview Guidelines)**

| **1** | **Warm-up & Introduction** | 5분 | 00:05 |
| --- | --- | --- | --- |

- 1. **인터뷰 목적, 내용 및 방식 관련 설명
     (Describe the purpose, content, and method of the interview)**
  2. **참여자의 일반적 특성 조사
     (Research general characteristics of participants)**
  3. **서면 동의서 작성
     (Complete written consent)**

| **2** | **담당 입원 환아의 환자안전사건에 대한 경험**  **(Experience with patient safety incident involving hospitalized children you were in charge of)** | 25분 | 00:30 |
| --- | --- | --- | --- |

**2-1 귀하가 경험한 담당 입원 환아의 환자안전사건은 무엇이었습니까?**

**What patient safety incident have you experienced in the hospitalized children you were in charge of?**

**2-2 가장 기억에 남는 환자안전사건은 무엇이었습니까?
 What was your most memorable patient safety incident?**

- 그 사건은 지금으로부터 얼마 전에 발생하였습니까?

How long ago was the event?

- 그 사건은 어떤 사고였습니까?

What kind of event was it?

1. 진단 관련(잘못된 진단, 진단 지연 등)

Diagnostic related (incorrect diagnosis, delayed diagnosis, etc.)

2. 약물, 수혈 관련(약물 부작용, 수혈 부작용)

Drug, transfusion-related (drug side effects, transfusion side effects)

3. 수술, 시술 관련(수술 출혈, 수술 천공 등)

Surgical, procedure-related (surgical bleeding, surgical perforation, etc.)

4. 감염 관련(수술부위 감염, 도뇨관 요로감염 등)

Infection-related (surgical site infections, catheterized UTIs, etc.)

5. 환자 케어 관련(낙상, 욕창, 자살 등)

Patient care related (falls, pressure ulcers, suicide, etc.)

6. 기타

Etc.

- 그 사건이 발생한 정황에 대해 말씀해주십시오.

Tell us about the circumstances of the incident.

- 그 사건의 가장 중요한 원인은 무엇이라고 생각합니까? 그 이유를 말씀해주십시오.

What do you think the most important cause of the incident was? Please tell us why.

- 그 사건에 의료진이나 병원의 과실이 있었다고 생각합니까? 그 이유를 말씀해주십시오.

Do you think there was any negligence by the healthcare provider or hospital in the incident? Please tell us why.

- 그 사건에 환아나 부모의 실수가 있었다고 생각합니까? 그 이유를 말씀해주십시오.

Do you think there was a child or parent error in the incident? Please tell us why.

| **3** | **입원 환아의 환자안전사건 이후 경험**  **(Experience after a patient safety incident in a hospitalized child)** | 15분 | 00:45 |
| --- | --- | --- | --- |

[가장 기억에 남는 환자안전사건 위주로 진행]
[focus on your most memorable patient safety incident].

**3-1 환아가 입은 위해는 어느 정도였습니까?
How much harm was done to the child?**

- 환아가 입은 위해의 정도는 어떠했는지? (자세하게 설명하도록)
 How much harm was done to the child? (Describe in detail)

- 해당 사고 이후 환아의 경과는? (자세하게 설명하도록)
 What has happened to the child since the incident? (Describe in detail)

**3-2 발생한 환자안전사건에 대해 환아, 보호자의 반응은 어떠하였습니까?
How did the child's and parents react to the patient safety incident that occured?**

- 해당 사고 이후 환아의 반응은?

How did the patient react after the incident?

- 해당 사고 이후 환아 부모의 반응은? (자세하게 설명하도록)

How did the parents react after the incident? (Describe in detail)

- 환아 또는 부모와 당신과의 관계에 있어 변화가 있었습니까?

Have there been any changes in your relationship with the child or parents?

**3-2 발생한 환자안전사건 이후 해당 근무부서, 병원에서 개선 활동 등 변화가 있었습니까?
Have there been any changes in your unit or hospital since the patient safety incident, such as improvement activities?**

- 해당 사고 이후 부서의 변화가 있었습니까? (자세하게 설명하도록)

Have there been any changes in the department since the incident? (Describe in detail)

- 해당 사고 이후 의료기관에서의 변화가 있었습니까? (자세하게 설명하도록)

Have there been any changes in the hospital since the incident? (Describe in detail)

| **4** | **입원 아동의 환자안전사건 예방 및 개선을 위해 필요한 부분**  **(What is needed to prevent and improve patient safety incidents in hospitalized children?).** | 10분 | 00:55 |
| --- | --- | --- | --- |

**4-1 입원 아동의 환자안전사건 예방 및 개선과 관련하여 필요한 부분은 무엇입니까?**

**What is needed to prevent and improve patient safety incidents in hospitalized children?**

- 입원 아동의 환자안전사건 예방을 위해 가장 필요한 것은 무엇이라고 생각합니까?

What do you think is most needed to prevent patient safety incidents in hospitalized children?

- 입원 아동의 환자안전사건 개선과 관련하여 가장 시급한 사항은 무엇이라고 생각합니까?

What do you think is the most urgent issue regarding improving patient safety incidents for hospitalized children?

- 입원 아동의 환자안전사건 예방 및 개선과 관련하여 의료진, 그 중 특히 간호사의 역할은 무엇이라고 생각합니까?

What do you think is the role of medical staff, especially nurses, in preventing and improving patient safety incidents in hospitalized children?

- 입원 아동의 환자안전사건 예방 및 개선과 관련하여 부모의 역할은 무엇이라고 생각합니까?

What do you think is the role of parents in preventing and improving patient safety incidents in hospitalized children?

- 입원 아동의 환자안전사건 예방 및 개선과 관련하여 병원 측에 바라는 부분은 어떤 점입니까?

What do you expect from the hospital regarding prevention and improvement of patient safety incidents in hospitalized children?

- 입원 아동의 환자안전사건 예방 및 개선과 관련하여 전체 의료시스템, 의료 정책 등에 바라는 부분은 어떤 점입니까?

What do you expect from the overall healthcare system, healthcare policy in relation to preventing and improving patient safety incidents in hospitalized children?

| **5** | **추가질문과 답변**  **(Additional questions and answers)** | 5분 | 01:00 |
| --- | --- | --- | --- |

- 입원 아동의 환자안전사건 경험과 관련하여 추가적으로 말씀하시고 싶은 부분에 대해 자유롭게 말씀해주세요.

Please feel free to tell us anything else you would like to add regarding your experience with patient safety incidents involving hospitalized

children.
